# Energy Drink Administration in Combination with Alcohol Causes an Inflammatory Response and Oxidative Stress in the Hippocampus and Temporal Cortex of Rats

**DOI:** 10.1155/2016/8725354

**Published:** 2016-03-16

**Authors:** Alfonso Díaz, Samuel Treviño, Jorge Guevara, Guadalupe Muñoz-Arenas, Eduardo Brambila, Blanca Espinosa, Albino Moreno-Rodríguez, Gustavo Lopez-Lopez, Ulises Peña-Rosas, Berenice Venegas, Anabella Handal-Silva, José Luis Morán-Perales, Gonzalo Flores, Patricia Aguilar-Alonso

**Affiliations:** ^1^Facultad de Ciencias Químicas, Benemérita Universidad Autónoma de Puebla, 72570 Puebla, PUE, Mexico; ^2^Departamento de Bioquímica, Facultad de Medicina, Universidad Nacional Autónoma de México, 04510 Ciudad de México, DF, Mexico; ^3^Departamento de Bioquímica, Instituto Nacional de Enfermedades Respiratorias, 14080 Ciudad de México, DF, Mexico; ^4^Departamento de Biología y Toxicología de la Reproducción, Instituto de Ciencias, Benemérita Universidad Autónoma de Puebla, 72570 Puebla, PUE, Mexico; ^5^Laboratorio de Neuropsiquiatría, Instituto de Fisiología, Benemérita Universidad Autónoma de Puebla, 72570 Puebla, PUE, Mexico

## Abstract

Energy drinks (EDs) are often consumed in combination with alcohol because they reduce the depressant effects of alcohol. However, different researches suggest that chronic use of these psychoactive substances in combination with alcohol can trigger an oxidative and inflammatory response. These processes are regulated by both a reactive astrogliosis and an increase of proinflammatory cytokines such as IL-1*β*, TNF-*α*, and iNOS, causing cell death (apoptosis) at the central and peripheral nervous systems. Currently, mechanisms of toxicity caused by mixing alcohol and ED in the brain are not well known. In this study, we evaluated the effect of chronic alcohol consumption in combination with ED on inflammatory response and oxidative stress in the temporal cortex (TCx) and hippocampus (Hp) of adult rats (90 days old). Our results demonstrated that consuming a mixture of alcohol and ED for 60 days induced an increase in reactive gliosis, IL-1*β*, TNF-*α*, iNOS, reactive oxygen species, lipid peroxidation, and nitric oxide, in the TCx and Hp. We also found immunoreactivity to caspase-3 and a decrease of synaptophysin in the same brain regions. The results suggested that chronic consumption of alcohol in combination with ED causes an inflammatory response and oxidative stress, which induced cell death via apoptosis in the TCx and Hp of the adult rats.

## 1. Introduction

Alcohol abuse causes serious social and economic problems as well as several pathological consequences [1, 2]. Studies indicate that alcohol abuse causes the death of 2.5 million people every year [3]. The Center for Disease Control and Prevention ranked alcohol abuse as the third leading cause of preventable death [4]. In recent years, there has been an increase in alcohol-related problems, especially amongst young drinkers. This is a major concern, because the early onset of alcohol abuse is a major risk factor in the onset of metabolic and degenerative disorders [5]. Furthermore, the situation becomes worse as the consumption of other substances with alcohol becomes increasingly more common. Recently, it has been shown that the energy drink (ED) is mainly accompanied with alcohol [6, 7]. These beverages contain high concentrations of caffeine, taurine, and carbohydrates (sucrose and glucose) with B-complex vitamins. These drinks have been marketed as a way to provide increased alertness [8–10]. Some concerns regarding the combined use of alcohol and EDs have arisen because recent studies indicate that the consumption of EDs induces more alcohol consumption, owing to the reduction of the depressant effects of the alcohol by the EDs [11] and causing serious physiological problems [1, 5, 6, 12].

The evidence indicates that acute consumption of EDs decreases motor and cognitive disturbances induced by alcohol intoxication. This is due to the stimulating action produced by the constituents of the ED on the brain. It has been demonstrated that chronic alcohol consumption induces death of neurons in cognitive related brain regions, such as the hippocampus and the cerebral cortex of adult rats. But reports about toxic effects on brain by consumption of these mixed drinks do not exist. Some reports indicate that alcohol causes oxidative stress in hippocampal neurons provoking an inflammatory response, which is regulated by reactive astrogliosis and increased proinflammatory cytokines such as IL-1*β*, TNF-*α*, and iNOS. Consequently, this event triggers death in neurons, evidenced by the activation of caspase-3 and reduced synaptophysin concentration, especially in the hippocampus and brain cortex of adult rats [13, 14].

Although it has been found that caffeine, vitamin B12, and taurine separately exert neuroprotective effects in the hippocampus and cortex of rats, how an ED can affect the brain's structure and functions when it is combined with alcohol is unknown. The objective of this study was therefore to demonstrate the effect of the combination of EDs and alcohol on oxidative stress and inflammatory responses in the hippocampus and the temporal cortex of rats.

## 2. Materials and Methods

### 2.1. Animals

Three-month-old adult male Wistar rats (230–250 g, *n* = 32) were provided by the animal housing “Claude Bernard” from the Autonomous University of Puebla. The rats were housed in polycarbonate cage bottoms, in a controlled climate and regulated light with 12 : 12-h day-night cycles with free access to food and water “*ad libitum.*” All procedures described in this study are in accordance with the Guide for the Care and Use of Laboratory Animals of the Mexican Council for Animal Care NOM-062-ZOO-1999. Every effort was made to minimize the number of animals used and to ensure minimal pain and/or discomfort to the animals.

### 2.2. Protocol of the Experimental Groups

Four experimental groups were designed for the study (8 rats/group): (1) vehicle or control (only drinking water), (2) energy drink (ED) (7.5 mL/kg), (3) alcohol (2.5 g/kg), and (4) alcohol + ED (2.5 g/kg + 7.5 mL/kg). All substances were administered orally (beverage) for 60 days at 10 am. The doses for the treatment were chosen or calculated on the basis of previous reports [15–19]. The animals were housed individually and had free access to water and food. The energy drink (most popularly commercially available) has the following composition: 11 g sugar, 400 mg taurine, 50 mg caffeine, and Vitamin-B complex (8 mg niacin, 2 mg pantothenic acid, 2 mg pyridoxine, 60 *μ*g riboflavin, and 2 *μ*g cobalamin) in 250 mL.

### 2.3. Serum Ethanol Determinations

In the next treatment (day 60), samples were drawn by cardiac puncture utilizing Becton & Dickinson Vacutainer blood collection tubes containing additives as sodium fluoride (15.0 mg) and potassium oxalate (12.0 mg [100/sp, 1000/ca]), according to manufacturer's instructions. Serum ethanol content levels were determined using the Dade International (formerly DuPont) ACA IV method. All reagents were supplied by the instrument's manufacturer. The ACA IV was operated, maintained, and calibrated as specified by the manufacturer's instructions. The calibration range was 0 through 300 mg/dL.

### 2.4. Histological Examination

Following treatment, the rats (*n* = 4/group) were anesthetized with sodium pentobarbital (40 mg/kg, ip) and then perfused with 200 mL of 4% paraformaldehyde. The brains were removed and postfixed in the same fixative solution for 48 h and then embedded in paraffin. Coronal 5 *μ*m thick sections were taken from each brain at the level of the anterior temporal area, approximately −3.8 to −6.8 mm from the bregma.

### 2.5. Immunohistochemistry

Paraffin was removed from the sections (5 *μ*m thick) and they were rehydrated according to conventional histological techniques [20, 21]. The nonspecific binding sites were blocked by incubating in 2% IgG-free bovine serum albumin (BSA, Sigma). Afterwards, specimens were incubated with 0.2% Triton X-100. The sections were incubated overnight at 4 to 8°C with primary antibodies: glial fibrillary acidic protein (GFAP) (1 : 500, Dako A/S, Denmark) to mark astrocytes, inducible nitric oxide synthase (iNOS), synaptophysin, and caspase-3 all the antibodies, 1 : 100 (Santa Cruz Biotechnology Inc., CA, USA), which were determined, were observed by anti-rabbit or anti-mouse fluorescein isothiocyanate- (FITC-) labelled secondary antibodies (1 : 100, Jackson ImmunoResearch Laboratories Inc., PA, USA). Slides of GFAP were counter-stained with VectaShield (Vector Labs., CA, USA) and slides of iNOS synaptophysin and caspase-3 were mounted with VectaShield containing 4′,6-diamidino-2-phenylindole (DAPI) (Vector Labs., CA, USA) for nuclei staining. Photomicrographs were taken using a fluorescence microscope (Leica Microsystems GmbH, Wetzlar, Germany) and projected with a Leica IM1000 version 1.20 release-9 computer based program (Imagic Bildverarbeitung AG, Leica Microsystems, Heerbrugg, Switzerland). The number of immunoreactivity cells of GFAP was quantified in the TCx and the CA1 subfield of the hippocampus (Hp). The criteria that define reactive astrocytes comprise GFAP immunoreactivity. All counting procedures were performed by double-blind method by experts in morphology.

### 2.6. ELISA to Quantify Proinflammatory Cytokines (IL-1*β* and TNF-*α*)

Following treatment, the animals were decapitated (*n* = 4/group), their brains were immediately removed and washed in cold SSI, and the Hp and TCx were dissected. These were homogenized in 3 mL of 0.1 M, pH 7.4, cold Phosphate Buffered Saline (PBS). Homogenates were centrifuged at 12,500 rpm at 4°C. The supernatant was obtained and stored at −70°C until used for protein and proinflammatory cytokine measurements [22, 23].

The concentrations of IL-1*β* and TNF-*α* in homogenates of the TCx and Hp of rats were quantified by a sandwich immunoassay procedure, as specified in the kit protocols (R&D Systems, Minneapolis, MN, USA). Samples were placed into polyclonal antibody precoated wells and the immobilized antibody bound the interleukin in question. After washing away any unbound substances, an enzyme-linked specific antibody was added to the wells. After washing, a substrate solution was added to the wells. The enzyme reaction yielded a blue product that turned yellow when stop solution was added. Samples were read in a microplate reader at a wavelength of 450 nm. The intensity of the measured color was in proportion to the amount of the cytokine. The lower detection limits of these ELISA data are in the range of 10 to 15 pg per mg of protein.

### 2.7. Measurement of Nitric Oxide (NO)

Nitrite (NO_2_^−^), a stable breakdown product of NO, was measured with the Griess Reagent System (Promega, Madison, WI) [22]. Absorbance was measured with a 540 nm filter in a Beckman spectrophotometer. Results were expressed as micromoles of nitrite per milligram of protein (*μ*M of NO_2_^−^/mg of protein).

### 2.8. Assay of Lipid Peroxidation

The formation of lipid-soluble fluorescent compounds was measured using the established method described previously [17, 20]. 4 mL of a chloroform-methanol mixture (2 : 1, v/v) was added to aliquots of 1 mL from previous homogenates. Samples were stirred and placed on ice for 30 min in the dark. The upper phase was discarded and fluorescence of the chloroform phase was determined at 370 nm excitation and 430 nm emission wavelengths in a PerkinElmer LS50-B luminescence spectrometer. The sensitivity of the equipment was adjusted to a fluorescent signal of 140 fluorescence units (FU) with a standard quinine solution (0.001 mg/mL quinine in 0.05 M H_2_SO_4_). The evaluation of the lipid peroxidation was the same as that used for the assay of the reactive oxygen species. The results were expressed as relative fluorescence units (RFU) per milligram of protein [23].

### 2.9. Assay of Reactive Oxygen Species

Cellular ROS were evaluated using 5 *μ*L of homogenized tissues, which were diluted in 9 vol. of 40 mM TRIS plus HEPES buffer, and then incubated with 5 *μ*M 2′7′-dichlorodihydrofluorescein diacetate (DCFH-DA). The samples were incubated for 1 h at 37°C under constant shaking before the fluorescence signals were determined in a PerkinElmer LS50-B luminescence spectrometer at 488 nm excitation and 525 nm emission wavelengths. Values were obtained by interpolating the readings with a 2′7′-dichlorofluorescein (DCF) standard curve (Sigma-Aldrich). The results were expressed as nanomoles of DFC formed per milligram of protein per minute [23].

### 2.10. Statistical Analysis

The results were expressed as the mean ± standard error (SE) for all experiments. Statistical analyses were done using analysis of variance, and multiple comparisons were made using Bonferroni's post hoc test or the one-way ANOVA, considering *P* < 0.05 as significant. GraphPad Prism 5.0 was also used.

## 3. Results

### 3.1. Serum Ethanol Determinations

**Figure 1:**
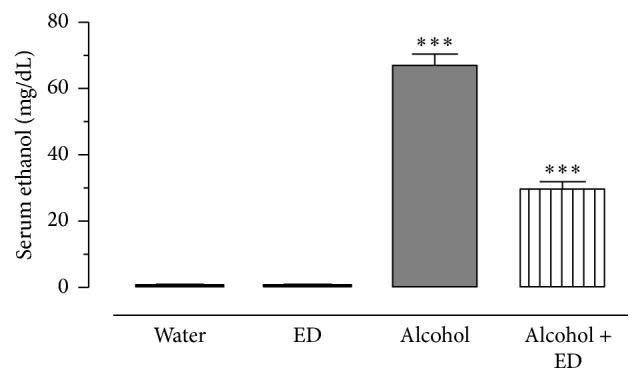
Serum ethanol determinations in ethanol injected rats for 60 days. The blood alcohol concentration, the group injected with ethanol only (2.5 g/kg), and the group treated with alcohol plus ED for 60 days. The values show the mean ± SE (*n* = 8) (Student's *t*-test; ^*∗∗∗*^ *P* < 0.001 as significant).

In Figure 1, analytical results for alcohol determinations in serum from different worked groups are represented. As it was expected, water and ED groups did not present detectable levels of ethanol, whereas in the alcohol group (2.5 g/kg) the mean levels of serum ethanol at end of study were of 67 mg/dL. On the other hand, in alcohol + ED group (2.5 g/kg + 7.5 mL/kg) alcohol levels showed correspondence to 30 mg/dL, which suggest that ED combined, enhances the clearance pattern avoiding toxicological effects for alcohol consumption.

### 3.2. GFAP Immunoreactivity

In order to understand the effects caused by the combination of alcohol and ED on the inflammatory response in the TCx and Hp of rats, the immunoreactivity to GFAP and the concentration of IL-1*β* and TNF-*α* were evaluated in these brain regions.

**Figure 2:**
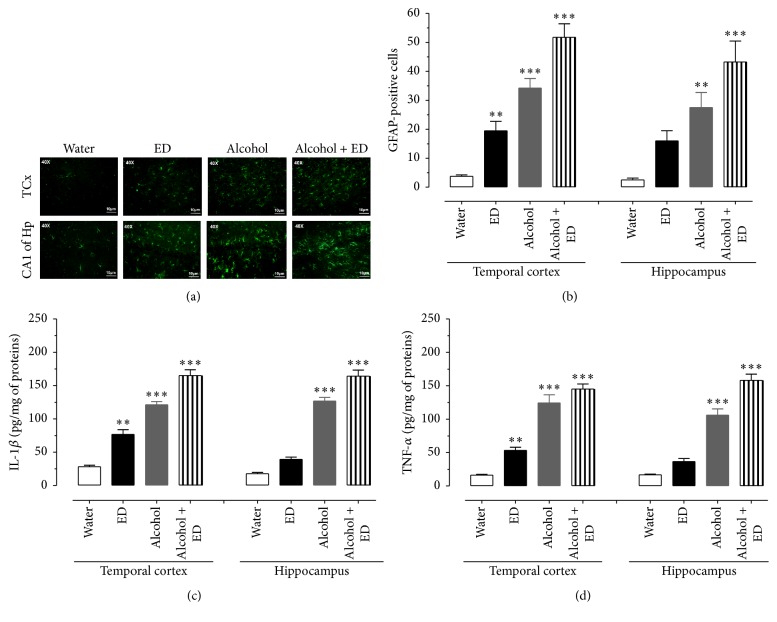
Mixing alcohol and ED caused inflammatory responses in temporal cortex and hippocampus of rat. The photomicrographs show the immunoreactivity to GFAP (green colour) of the TCx and CA1 subfield of the Hp of rat. (a) Control group (water), ED, alcohol, and the mixture of alcohol and ED. (b) Number of GFAP-positive cells of treated groups. (c) and (d) show the concentrations of IL-1*β* and TNF-*α*, respectively, in TCx and Hp of treated groups. The values show the mean ± SE (*n* = 4) (one-way ANOVA with Bonferroni's post hoc test; ^*∗∗*^ *P* < 0.01; ^*∗∗∗*^ *P* < 0.001).

Figure 2(a) shows the immunoreactivity to GFAP (green color) in the four study groups. The results indicate that, for animals treated with ED, alcohol, and alcohol plus ED, the immunoreactivity to GFAP in the TCx and CA1 subfield of the Hp increased, comparing the photomicrographs to those of the control group. In particular, the increased GFAP immunoreactivity in the alcohol plus ED group was distributed in greater proportion compared to the groups treated separately with alcohol or ED. The quantitative data for GFAP-positive cells in the TCx show that the administration of alcohol mixed with energy drinks showed a 12-fold increase in the number of reactive astrocytes with respect to the control group (Figure 2(b)). Likewise, the mixed beverages group produced an astrogliosis greater than that produced by the ED and alcohol groups (one-way ANOVA with significance of *P* < 0.05).

Similarly, the number of GFAP-immune positive cells in the CA1 subfield of the Hp indicates that consumption of alcohol mixed with EDs induces greater immunoreactivity to GFAP, when compared to the consumption of water, EDs, and alcohol alone (Figure 2(b)) (one-way ANOVA with significance of *P* < 0.05).

### 3.3. Evaluation of Proinflammatory Cytokines, iNOS Immunoreactivity, and NO Levels

**Figure 3:**
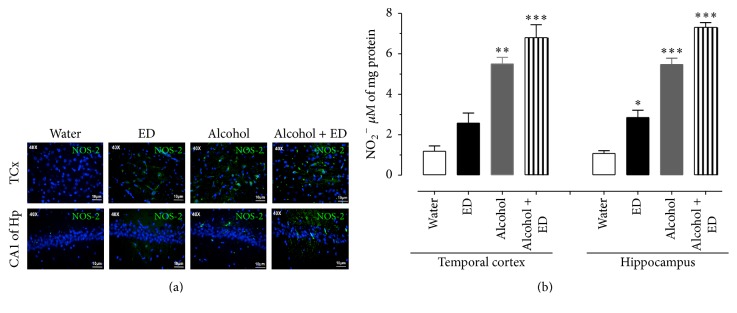
Effect of the combination of alcohol and ED on iNOS immunoreactivity and nitrite concentration in temporal cortex and hippocampus of rat. The photomicrographs show the immunoreactivity to iNOS (green colour) of the TCx and CA1 subfield of the Hp of rat. (a) Control group (water), ED, alcohol, and the mixture of alcohol and ED. (b) shows the concentration of nitrites in TCx and Hp of treated groups. The values show the mean ± SE (*n* = 4) (one-way ANOVA with Bonferroni's post hoc test; ^*∗*^ *P* < 0.05; ^*∗∗*^ *P* < 0.01; ^*∗∗∗*^ *P* < 0.001).

The concentration of cytokines which were determined from the homogenates of the TCx and Hp of rats after 60 days of consumption of water, ED, and alcohol as well as their combination is shown in Figures 2(c) and 2(d). The levels of IL-1*β* in the TCx and Hp clearly show that animals that consumed a combination of alcohol and ED recorded the highest concentration of IL-1*β*, both in the TCx and in Hp, with respect to the other groups (one-way ANOVA with significance of *P* < 0.05). Similarly, TNF-*α* levels from rats treated with alcohol and ED were also significantly higher in both brain regions of interest with respect to the control, ED, alcohol treated groups, respectively (one-way ANOVA with significance of *P* < 0.05). To determine the effect of the consumption of alcohol and ED on the immunoreactivity to iNOS and the concentration of nitrite (stable metabolite from NO) in the TCx and Hp, the tissues were examined at 60 days of administration. We observed that the anti-iNOS antibody (green color) showed a more intense reaction to the treatment with alcohol and ED, being distributed uniformly in the TCx and CA1 subfield of the Hp, whereas the groups treated only with alcohol, ED, and water showed a lower immunoreactivity to iNOS in the same areas of the brain (Figure 3(a)). NO concentration was evaluated indirectly by measuring nitrite (NO_2_^−^) from the homogenates of the TCx and Hp of the different groups.  Figure 3(b) shows that NO_2_^−^ levels in the TCx and Hp of the alcohol with ED group were significantly higher compared with the control group (558 and 580%, resp.); also NO_2_^−^ levels in separate administration of ED (203 and 156%) and alcohol (29 and 28%) in relation to control group (one-way ANOVA with significance of *P* < 0.05).

### 3.4. Oxidative Stress

**Figure 4:**
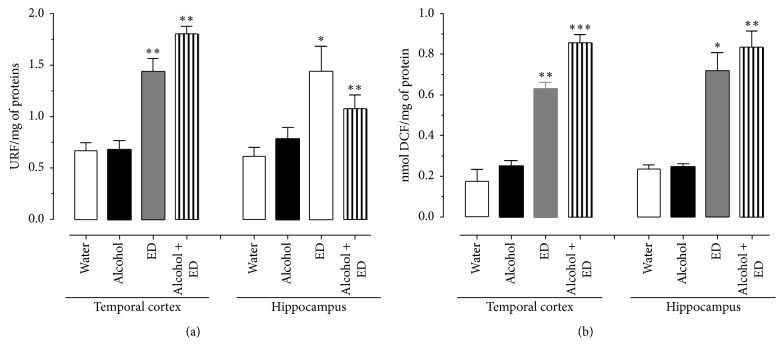
Effect of the combination of alcohol and ED on lipid peroxidation (a) and formation of reactive oxygen species (b) in the rat temporal cortex and hippocampus of rat. Control group (water), ED, alcohol, and the mixture of alcohol and ED. The values show the mean ± SE (*n* = 4) (one-way ANOVA with Bonferroni's post hoc test; ^*∗*^ *P* < 0.05; ^*∗∗*^ *P* < 0.01; ^*∗∗∗*^ *P* < 0.001).

The data obtained from lipid peroxidation measurements are shown in Figure 4(a). The results indicate that animals which drank the combination of alcohol and ED presented the highest lipid peroxidation levels in the TCx and Hp (168 and 180%, resp.) compared to the control, while the ED and alcohol groups showed levels of 133 and 119% and 25 and 17%, respectively (one-way ANOVA with significance of *P* < 0.05).

The amount of 2′,7′-dichlorodihydrofluorescein found in the TCx and Hp tissues is shown in Figure 4(b). Statistical analysis indicates that the largest significant difference, in relation to control group, corresponds to the treatment with mixed drinks (264 and 260%), while the ED group shows 203 and 232% and alcohol group shows only 37 and 17%, respectively.

### 3.5. Caspase-3 and Synaptophysin Immunoreactivity

**Figure 5:**
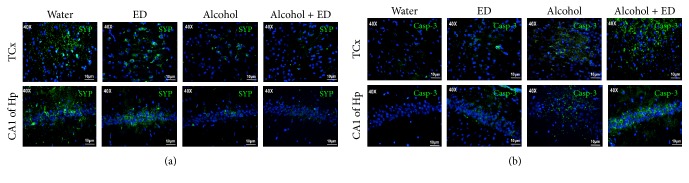
The combination of alcohol and ED causes an increase of caspase-3 and synaptophysin immunoreactivity in temporal cortex and hippocampus of rat. The photomicrographs show the immunoreactivity to synaptophysin (a) and caspase-3 (b) (both marked in green) in the TCx and Hp of rat. Control group (water), ED, alcohol, and the mixture of alcohol and ED.

The results observed here strongly indicate death of neurons caused by alcohol consumption combined with ED; so we evaluated synaptophysin and active caspase-3 immunoreactivity. It is observed in the photomicrographs of Figures 5(a) and 5(b) that the TCx and CA1 subfield of the Hp of the control and ED groups show cells with greater immunoreactivity for synaptophysin (green color) and low marks for caspase-3 (green color), respectively. In contrast, the alcohol group and mixed drinks group show an intense reactivity for caspase-3 and a decrease in the immunoreactivity for synaptophysin. Particularly, the animals treated with the combination of alcohol and ED presented a greater intensity to caspase-3 and a low reactivity for synaptophysin. This suggests that mixed drinks exacerbate the death in neurons of the TCx and CA1 of the Hp of rats.

## 4. Discussion

In this study we administered alcohol in combination with ED in rats for 60 days. We found that the combination of these two drinks causes an inflammatory response, oxidative stress, and cell death on temporal cortex and hippocampus of rats. These findings demonstrate the negative and serious effects of alcohol consumption in combination with ED.

Since the introduction of EDs to the US in 1997, their consumption alone and in combination with alcohol has been increased [9, 24]. These products essentially combine performance-enhancing ingredients (taurine, ginseng, green tea extract, and/or B-complex vitamins), which are characterized by psychostimulant effects in humans and animals [24, 25]. The alcohol is widely recognized as a CNS depressant, when administered in high doses (greater than 2 g/kg) [26, 27]. The neurobiological mechanism to explain the interaction between alcohol and the ED is not clear yet. Reports indicate that alcohol, caffeine, and taurine (two compounds of ED) interact with the glutamatergic system, which increases the release of glutamate and cytotoxicity [28, 29]. Furthermore, caffeine is a competitive antagonist of adenosine receptors, whereby it can exacerbate the dopaminergic system [30] and help improve the negative effect of alcohol-depressive in animals [31]. A situation that had not been demonstrated by the chronic consumption of alcohol and ED in the temporal cortex and hippocampus of rats; brain regions that are highly susceptible to these death neurons process.

The inflammatory response in the brain involves reactive astrocytes and the production of various proinflammatory mediators, such as proinflammatory cytokines and deleterious free radicals, which are associated with stress-activated signal transduction pathways, leading to death in neurons [32, 33]. These disorders display an accumulation of derivative products from the increase of oxidative stress, leading to widespread damage of lipids and protein. In this work it was observed that the combination of alcohol and ED induces increased astrogliosis in the temporal cortex and hippocampus compared with the administration of only alcohol or ED. It has been demonstrated that long term, chronic alcohol consumption causes reactive astrocytosis and enhances the release of proinflammatory mediators, mainly in brain regions such as the temporal cortex and hippocampus [34, 35]. Our results clearly show the presence of reactive astrogliosis induced by alcohol, besides the increase in IL-1*β* and TNF-*α*, in the two brain regions of study.

Furthermore, the effect of ED on neuroinflammation has not been described, although Kang et al. [33] reported that consuming caffeine in high concentrations causes an inflammatory response and death in neurons of neonatal rats and in cell cultures. Nevertheless, in the brains of animals treated only with ED, low immunoreactivity to GFAP was observed (see Figure 2(a)).

We can further add that the group given alcohol and ED showed higher immunoreactivity to GFAP both in the temporal cortex and in hippocampus with respect to the other experimental groups. Possibly, a synergistic effect between ingredients of the energy drink with alcohol is able to exacerbate the release of glutamate; however, this is still unclear, suggesting that reactive astrogliosis and the release of proinflammatory factors such as IL-1*β* and TNF-*α* are a reflex of the growing inflammatory response in the temporal cortex and hippocampus. Cytokines are considered to be regulators of the intensity and duration of the inflammatory process [36, 37]. These promote the activation of various signaling pathways in response to cellular stress, including iNOS, responsible for the increasing concentrations of NO, which reach toxic levels and lead to the development of oxidative stress and neuronal death* in vitro* [38, 39]. Therefore, we studied the effect of the combination of alcohol and ED on the immunoreactivity of iNOS and the concentration of NO_2_^−^. The results indicate that the administration of both drinks together causes an increase in iNOS immunoreactivity in temporal cortex and in the CA1 region of the hippocampus. Administration for 60 days could promote chronic glial activation accompanied by the increased release of proinflammatory cytokines such as IL-1*β* and TNF-*α*, thus triggering proliferation of glial cells [40]. Furthermore, regulating the expression of iNOS in astrocytes produces high concentrations of NO which facilitate the generation of oxidative stress and neuronal dysfunction [39]. The alcohol-induced inflammatory response, together with the ED, could be responsible for the NO production by iNOS activity, exacerbating brain inflammation [38], because NO is an important source of ROS, which contributes to oxidative stress process and death in neurons [18]. As shown in our results, the group given alcohol and ED presented a significant increase in the formation of free radicals and lipid peroxidation, compared with the control group and the group treated with ED only. For the group treated with alcohol only, the levels of reactive oxygen species and lipid peroxidation do not show a significant difference with respect to the interest group (alcohol with ED), suggesting that alcohol alone, and not the consumption of energy drinks alone, causes neurotoxic effects. Therefore, it is necessary to make the combination of alcohol plus ED to trigger oxidative stress and an inflammatory response. This consequently contributes to death in neurons of the temporal cortex and hippocampus.

Thus, we evaluated death in cells as to be caused by the combination of alcohol and ED in the temporal cortex and hippocampus of rats, using immunoreactivity for synaptophysin and caspase-3. Synaptophysin is a marker of neuronal function, while caspase-3 indicates the death of neurons by the apoptotic pathway.

**Figure 6:**
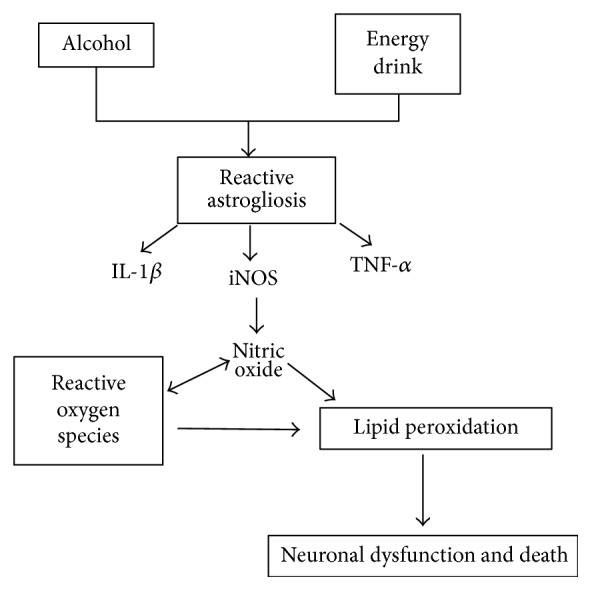
Proposed mechanisms of toxicity, which causes the mixture of alcohol and ED in the temporal cortex and hippocampus of rats.

It has been reported that chronic alcohol consumption in rats induces death in neurons by apoptosis in different hippocampal and cerebral cortex regions. This was demonstrated by the increase in immunoreactivity of caspase-3 [13, 26, 41]. With regard to EDs, there are no studies that report damage on neurons caused by chronic consumption of these beverages. Only one study suggests that consuming caffeine in high concentrations can cause neuronal death [33]. In this paper, the largest immunoreactivity of caspase-3 and minimal reactivity to synaptophysin in the hippocampus and temporal cortex of the animals treated with alcohol and ED were observed at 60 days when compared to the control group and ED. This indicates that chronic consumption of ED exacerbates death neurons via apoptosis induced by alcohol in rats. In summary, this study demonstrates for the first time that chronic consumption of alcohol in combination with ED induces an inflammatory response, oxidative stress, and neuronal death in the temporal cortex and hippocampus of rats (see Figure 6). In addition, it was also demonstrated that a daily intake of energy drinks can cause neuroinflammation on the regions studied. In this regard, it is necessary to implement strategies of prevention and provide information to consumers of these drinks, concerning the risks of neuronal death and serious brain damage.
